# Targeted delivery of TLR3 agonist to tumor cells with single chain antibody fragment-conjugated nanoparticles induces type I-interferon response and apoptosis

**DOI:** 10.1038/s41598-019-40032-8

**Published:** 2019-03-01

**Authors:** Isabell Schau, Susanne Michen, Alexander Hagstotz, Andreas Janke, Gabriele Schackert, Dietmar Appelhans, Achim Temme

**Affiliations:** 1 Department of Neurosurgery, Section Experimental Neurosurgery and Tumor Immunology, University Hospital Carl Gustav Carus, TU Dresden, Fetscherstraße 74, 01307 Dresden, Germany; 2 Leibniz Institute of Polymer Research Dresden e.V., Mailbox 120411, 01069 Dresden, Germany; 3 German Cancer Consortium (DKTK), Dresden, Germany; German Cancer Research Center (DKFZ), Heidelberg, Germany

## Abstract

Application of Toll-like receptor (TLR) agonists is a promising approach to treat cancer. In particular, nucleic acid-based TLR agonists such as short ssRNA and dsRNA molecules, which activate endosomal TLRs, can be delivered to tumors by use of nanoparticle delivery systems. However, such delivery systems bear unspecific side effects and poor pharmacokinetics. To overcome these limitations we developed a system for targeted delivery of a 50 bp dsRNA TLR3 agonist (Riboxxol) to treat PSCA-positive tumor cells, which consists of neutravidin conjugated to mono-biotinylated dsRNA and to humanized mono-biotinylated anti-PSCA single chain antibody derivative scFv(h-AM1)-BAP. The assembly of the components resulted in the formation of nanoparticle-like immunoconjugates designated Rapid Inducer of Cellular Inflammation and Apoptosis (RICIA). Anti-PSCA-RICIA exclusively delivered Riboxxol to PSCA-positive tumor cells as well as subcutaneous tumors. Uptake of anti-PSCA-RICIA induced a type I-interferon response and apoptosis in HEK-Blue^hTLR3/PSCA^ reporter cells and PSCA-positive HT1376 bladder cancer cells *in vitro*. No such effects were observed when using RICIA coupled to an unspecific control antibody or when using Riboxxol alone. Treatment of HT1376 xenografts in immune-deficient hosts with targeted delivery of TLR3 agonist did not induce adverse effects and only modestly inhibited tumor growth when compared to controls. These results suggest promising activation of innate immune response and apoptosis upon selective delivery of TLR3 agonists in tumor cells. Yet, further studies using syngeneic and orthotopic tumor models are needed to fully exploit the potential of RICIA immunoconjugates.

## Introduction

Molecules fundamental to eliciting an immune response in various cell types are Toll-like receptors (TLRs) and their cognate ligands. TLRs are a conserved family of pattern recognition receptors (PRRs) with 10 functional members in humans. They are expressed as homodimers and some as heterodimers, respectively, in a variety of immune cell types and other cell types^1,2^. TLRs are type I membrane proteins and contain an extracellular domain of leucine-rich repeats and an intracellular Toll/interleukin-1 receptor/resistance protein (TIR) domain. TLR1, 2, 4, 5, and 6 localize to the plasma membrane, while TLR3, 7, 8, and 9 are found in intracellular compartments of endosomes and lysosomes^1,2^.

Natural TLR ligands are pathogen-associated molecular patterns (PAMPs), or in other words, “danger signals”, normally not present in the host, but produced or expressed by microbiological pathogens such as bacteria and viruses. However, recent studies indicate that also some endogenous molecules released by dying or stressed cells, such as heat shock proteins (HSP), calreticulin and high mobility group box 1 (HMGB1) are also TLR ligands and are termed “damage-associated molecular patterns” (DAMPs)^3–6^.

TLRs are central to the induction of host protective adaptive immune responses. Vaccination against pathogens not only includes for instance microbiological or virus-derived products which might already contain PAMPs, but also include additional components, such as complete Freund´s adjuvant to boost activation of TLRs. Activation of TLRs of professional antigen-presenting cells, such as dendritic cells (DCs), is crucial for their maturation, homing into lymph nodes and upregulation of co-stimulatory receptors of the B7-protein family (CD80, CD86)^7^ and secretion of cytokines, which is all indispensable for activation of naïve T-cells^8^. Furthermore, ligands for TLRs are also involved in B-cell response by inducing B-cell proliferation and immunoglobulin isotype class switching^9^.

After binding of the respective ligand to TLR, signaling is mediated by one or more of four adaptor molecules: myeloid differentiation factor 88 (MyD88), TIR-domain-containing adapter-inducing interferon-β (TRIF), MyD88-adapter-like protein (MAL) and TRIF-related adaptor molecule (TRAM), which bind at the TIR domains of TLRs. TLR3 signals through TRIF which activates NF-κB, interferon regulatory factor (IRF) 3, IRF7 and other transcription factors to initiate a type I-interferon response and depending on the cell type lead to the release of further pro-inflammatory cytokines^1^. In mammals, anti-viral responses mediated by TLR3 binding to dsRNA include two reciprocal cellular programs which either induces cell survival with production of cytokines or apoptosis to eliminate infected cells. For instance, crosslinking of two TLR3s by dsRNA in the endosome of infected cells or antigen-presenting cells which have endocytosed viral debris or dsRNA, respectively, leads to recruitment of TRIF. The C-terminal region of TRIF then interacts with the receptor interacting protein 1 (RIP1), a serine-threonine protein kinase that can enhance survival through NF-κB and mitogen-activated protein kinase (MAPK) pathways, but also is capable of inducing apoptosis by recruiting Fas-associated death domain (FADD) and by activating caspase-8^10^.

TLR agonists used as single agent can efficiently eradicate tumors due their potent stimulation of innate and adaptive immunity as well as their effects on the tumor microenvironment. Yet, systemic application of TLR agonists as monotherapy or as combinatorial treatment with anti-cancer drugs bears the risk of accumulation in sinks like liver and lung and also unwanted activation of cells of the reticuloendothelial system. Hence, only two topically applied TLR agonists, Bacillus Calmette-Guérin (BCG), a putative TLR9 agonist, and imiquimod, a TLR7 agonist, have been approved for clinical use as monotherapy of non-muscle-invasive bladder cancer and of basal cell carcinoma, respectively^11^.

Conceptually, in contrast to other TLR agonists, systemic treatment of cancer patients with nucleic acid-based TLR agonists, such as ssRNA, dsRNA and CpG-oligonucleotides (ODNs) appears feasible since their cognate TLRs are located in the endosomal compartment of cells. Under physiological conditions such TLR agonists cannot easily cross membranes due to their size and negative net charge. So, several nanoparticle-delivery systems, for instance based on cationic polymeric carrier molecules or cationic liposomes, can be employed which enables cellular uptake and increases half-life of nucleic acids in the circulation^12^. Of note, such nanoparticles has been described to accumulate to a certain degree in tumor tissues due to enhanced permeability and retention effects, yet unspecific uptake in normal organs and cells is commonly observed^13^. One promising approach to minimize unwanted off-target effects of nanoparticle-delivery systems on normal tissues is the introduction of ligands that specifically bind to tumor cells, leading to the concept of targeted delivery.

In our study, we established a simple selective delivery system for TLR3 agonists to tumor cells using a recently described modular bio-conjugation system comprising neutravidin, mono-biotinylated single chain fragment variables (scFv) and mono-biotinylated TLR3 agonist Riboxxol-biotin, which belongs to a type of serum-stable dsRNA^14^. The consecutive assembly of the components resulted in nanoparticle-like structures which were termed as “Rapid Inducer of Cellular Inflammation and Apoptosis” (RICIA). As a model targetable surface receptor we focused on the prostate stem cell antigen (PSCA). PSCA represents an associated GPI-anchored cell surface antigen^15–18^ which is expressed in normal prostate-specific tissue and overexpressed in prostate cancer specimens including both, high-grade prostatic intraepithelial neoplasia and androgen-dependent/-independent tumors^18^. PSCA was also found to be expressed in prostate cancer metastases^19^ and in prostate-unrelated carcinomas such as renal clear cell carcinoma^20^, transitional cell carcinoma^21^, pancreatic adenocarcinoma^22^, glioblastoma, and HER2/neu-overexpressing breast cancer^23,24^. To be closer to a clinical application we furthermore humanized the PSCA-specific scFv(AM1)^25^ by engrafting the complimentary determining regions (CDRs) into human Ig germline genes. In this study we evaluate the selectivity and efficacy of anti-PSCA-RICIA for treatment of PSCA-positive tumor cells.

## Results

### Synthesis and functional characterization of humanized anti-PSCA scFv(h-AM1) and mono-biotinylated scFv(h-AM1)-BAP

**Figure 1:**
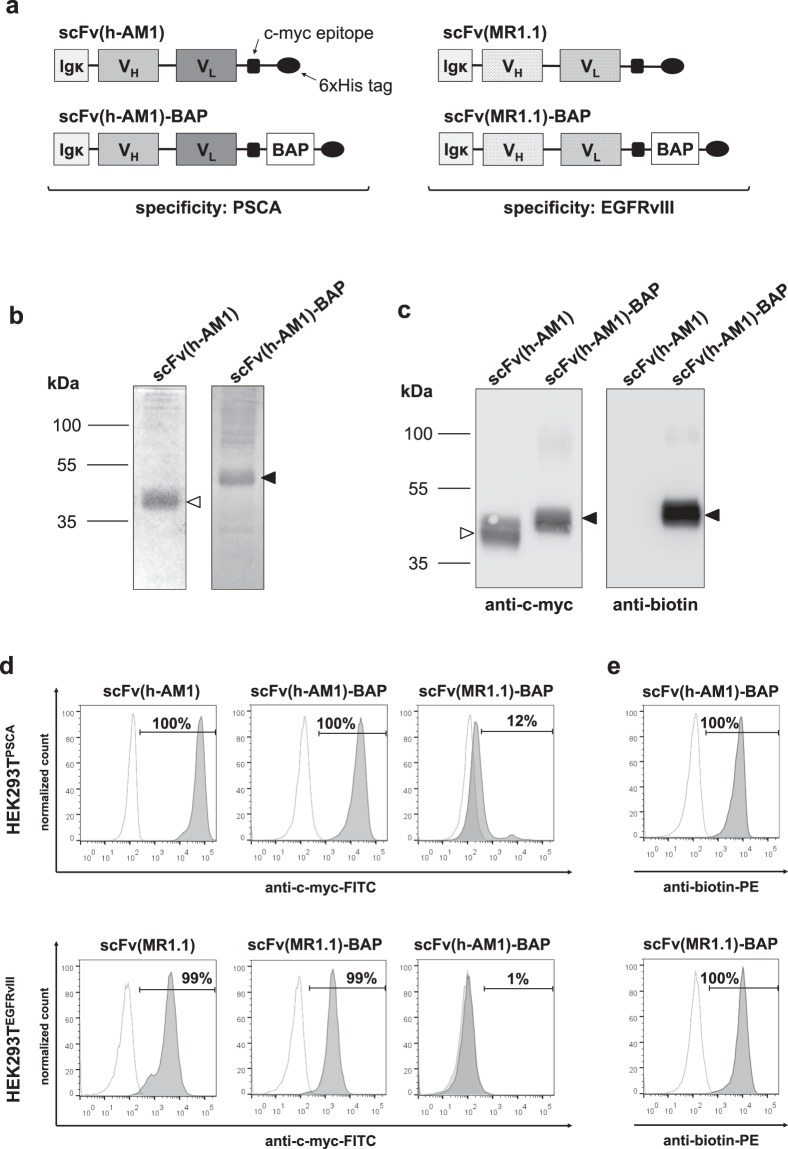
Generation and functional validation of parental scFvs and mono-biotinylated scFv-BAPs. (**a**) Schematic representation of PSCA- and EGFRvIII-specific parental scFv and scFv-BAP antibody constructs consisting of a Igκ-leader secretion signal, a variable heavy (V_H_) and a variable light (V_L_) chain, a C-terminal c-myc epitope and a 6x histidine (His) tag. A biotin acceptor peptide (BAP) was introduced to scFv-BAPs for site-specific enzymatic mono-biotinylation. (**b**) Purity of scFv(h-AM1) (open arrowhead) and scFv(h-AM1)-BAP (black arrowhead) was analyzed using Coomassie-stained polyacrylamide gel under reducing conditions. (**c**) Size of scFv(h-AM1) and scFv(h-AM1)-BAP via c-myc epitope and site-specific biotinylation of scFv(h-AM1)-BAP were assessed by Western blot analysis. The full-length blots/gels are presented in Supplementary Fig. 1. (**d**) Binding studies for functional characterization of scFvs and scFv-BAPs via the c-myc epitope and (**e**) for validation of mono-biotinylated scFv(h-AM1)-BAP and scFv(MR1.1)-BAP were undertaken using flow cytometry. Open histograms represent staining controls using only secondary antibodies.

Recently, we described a biotin-neutravidin-based modular system for antibody-mediated delivery of polycationic (poly)propyleneimine siRNA carrier molecules to tumor cells^26^. For targeted delivery of TLR3 agonist to tumor cells we sought to employ this platform technology and to couple biotinylated nucleic acid-based TLR agonists, in particular dsRNA, to tumor-specific antibodies via consecutive bio-conjugation to neutravidin. As a model tumor-antigen we choose PSCA, a surface protein which is found on several tumor entities^18–24^. For a potential clinical application we furthermore engineered an humanized scFv based on our recently described mono-biotinylated anti-PSCA scFv(AM1)-P-BAP^26^. For this we engrafted the complimentary determining regions (CDRs) of the murine scFv(AM1) in human germline Ig genes as outlined in the method section. We furthermore, exchanged the *Propionibacterium shermanii* transcarboxylase-derived biotin acceptor peptide (P-BAP) with a short 22 amino acid synthetic BAP to minimize potential immune responses of the host. As control for our studies we furthermore generated scFv(MR1.1)-BAP, which specifically binds a neo-epitope of the oncogenic epidermal growth factor receptor variant III (EGFRvIII). EGFRvIII is not present in healthy normal cells and the target cell lines used for this study, respectively. The structures of the resulting scFv-BAPs as well of the parental scFv are shown in Fig. 1a. All antibody constructs contained an N-terminal Igκ-leader for the extracellular secretion as well as a C-terminal c-myc epitope and 6x histidine (His) tag for detection and purification, respectively.

As demonstrated in Coomassie-stained polyacrylamide gels, the recombinant scFv(h-AM1)-BAP and scFv(h-AM1), the latter devoid of the BAP, were produced in high purity as full-length proteins, with bands at 50 kDa and 38 kDa, which is slightly higher than the respective calculated molecular masses (Fig. 1b). The increased molecular sizes of the antibody derivatives might be due to posttranslational glycosylation. Furthermore, besides the detection of the c-myc epitope, a biotin-specific antibody demonstrated the efficient site specific biotinylation of the humanized scFv(h-AM1)-BAP. As expected, the parental scFv(h-AM1) was devoid of biotin residues (Fig. 1c). Similar results were obtained with scFv(MR1.1)-BAP control antibodies (data not shown). The engrafting of the CDRs of the murine antibody into the framework region of human Ig germline genes did not affect the specificity of scFv(h-AM1) towards PSCA, as investigated in flow cytometry using HEK293T^PSCA^ cells (Fig. 1d). The EGFRvIII-specific control antibody bound to EGFRvIII-positive HEK293T^EGFRvIII^ cells but as expected not to HEK293T^PSCA^ cells. By using a PE-labeled biotin-specific antibody we furthermore demonstrated that the C-terminal biotin residue of scFv(h-AM1)-BAP and scFv(MR1.1)-BAP was accessible under native conditions, respectively (Fig. 1e). Unexpectedly, the murine scFv(AM1) acquired an improved affinity after humanization (Supplementary Tab. 1). A detailed comparison of the amino acid sequences of the murine and the humanized scFvs revealed subtle changes in potential O-glycosylation sites which in part might account for the improved affinity of the humanized anti-PSCA single chain antibody fragment (Supplementary Fig. 2).

### **Crosslinking of scFv(h-AM1) on HEK293T**^**PSCA**^**cells causes internalization of PSCA**

**Figure 2:**
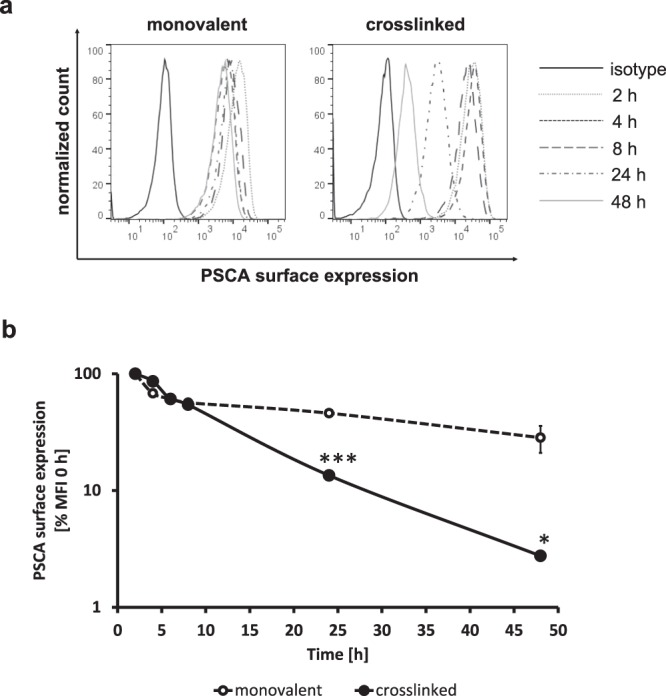
Crosslinking of scFv(h-AM1) causes significant PSCA receptor internalization on HEK293T^PSCA^ cells in a time-dependent manner. Internalization of PSCA after treatment with monovalent or crosslinked scFv(h-AM1) was assessed via flow cytometry. (**a**) Histograms of one representative experiment are shown. (**b**) Relative PSCA expression on HEK293T^PSCA^ cells after crosslinking was calculated based on mean fluorescence intensities (MFI) normalized to PSCA expression at 0 h. (*p < 0.05, ***p < 0.001).

Internalization of PSCA after crosslinking with nanoparticles is a prerequisite for the antibody-mediated delivery of TLR3 agonist. Therefore, it was of special interest whether crosslinking of at least two PSCA-molecules on the cell surface could induce the internalization of PSCA. Indeed, the crosslinking of scFv(h-AM1) with a biotinylated bivalent anti-c-myc-biotin antibody directed against the C-terminal c-myc epitope of antibody construct induced a significant time-dependent internalization of PSCA in HEK293T^PSCA^ cells, as assessed by staining with a tertiary anti-biotin-PE antibody (Fig. 2a). In contrast, the treatment of HEK293T^PSCA^ cells with monovalent scFv(h-AM1) antibodies alone did not induce a receptor internalization. The internalization of PSCA after crosslinking was calculated by the mean fluorescence intensity (MFI) for PSCA immunosignals (Fig. 2b). During the whole treatment period surface PSCA-expression levels did not completely disappeared after crosslinking as exemplified in representative flow cytometry histograms (Fig. 2a) which might be due to recycling of the receptor to the cell surface.

### Assembly and analysis of “Rapid Inducer of Cellular Inflammation and Apoptosis” (RICIA) immunoconjugates

**Figure 3:**
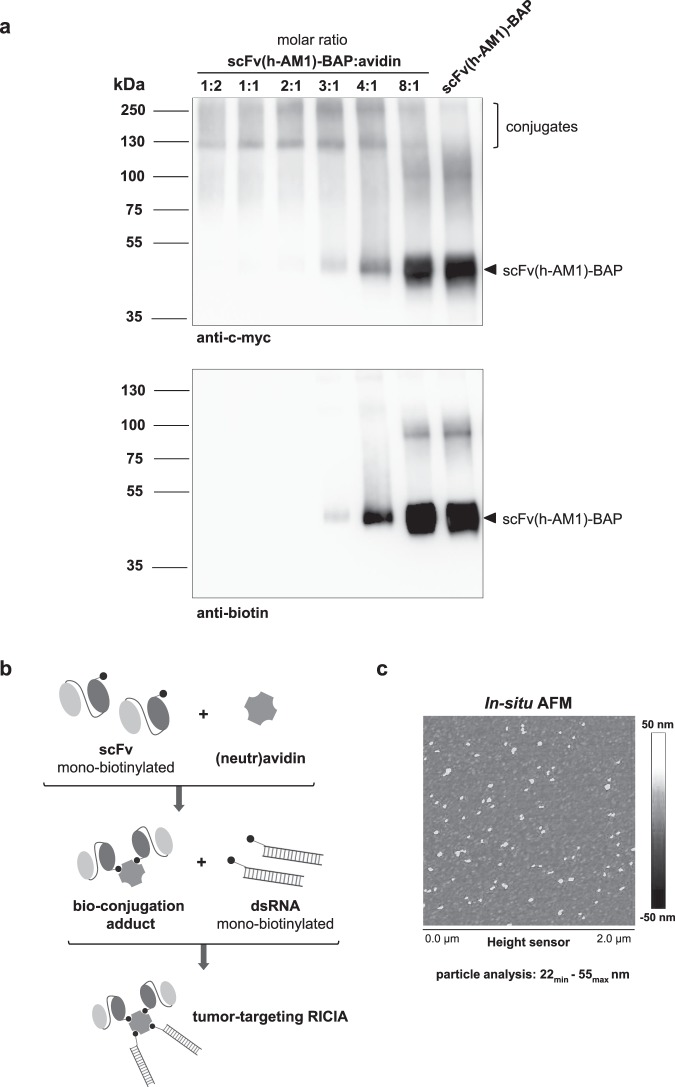
Generation of tumor-targeting scFv(h-AM1)-BAP-RICIA immunoconjugates. (**a**) Titration of scFv(h-AM1)-BAP against avidin in increasing molar ratios for the estimation of adequate conjugation conditions. Western blot analysis was performed to detect complexes via the c-myc epitope and free biotinylated scFv(h-AM1)-BAP. (**b**) Schematic representation of the successive conjugation of tumor-targeting RICIA. (**c**) Size of assembled scFv(h-AM1)-BAP-RICIA immunoconjugates was investigated using *in situ* atomic force microscopy (AFM).

The proper conjugation of biotinylated scFv(h-AM1)-BAPs to (neutr)avidin was assessed by gel shift assays. Here, a constant amount of scFv(h-AM1)-BAP was incubated with decreasing numbers of avidin molecules, resulting in increased molar ratios of scFv(h-AM1)-BAP to avidin (Fig. 3a). The scFv(h-AM1)-BAP/avidin conjugates were detected at molecular masses of approximately 120 kDa and 180 kDa via the C-terminal c-myc epitope of the scFv(h-AM1)-BAPs. Noteworthy, the conjugation with avidin essentially depleted the pool of free biotinylated scFv(h-AM1)-BAP molecules at scFv(h-AM1)-BAP to avidin ratio of 1:1 to 4:1 indicating an efficient conjugation. The experiments which were performed with scFv(MR1.1)-BAP revealed similar results (data not shown).

To produce immunoconjugates delivering TLR3 agonist to target cells with cognate receptor expression neutravidin was consecutively bio-conjugated to mono-biotinylated scFv(h-AM1) and mono-biotinylated Riboxxol as depicted in Fig. 3b. Subsequent *in situ* atomic force microscopy (AFM) was done after 8 h of fabrication (Fig. 3c). The diameters of immunoconjugates were found in the range of 22–55 nm.

### Anti-PSCA-RICIA specifically delivers 50 bp dsRNA TLR3 agonist into PSCA-expressing cells and induces a type I-interferon response and apoptosis

**Figure 4:**
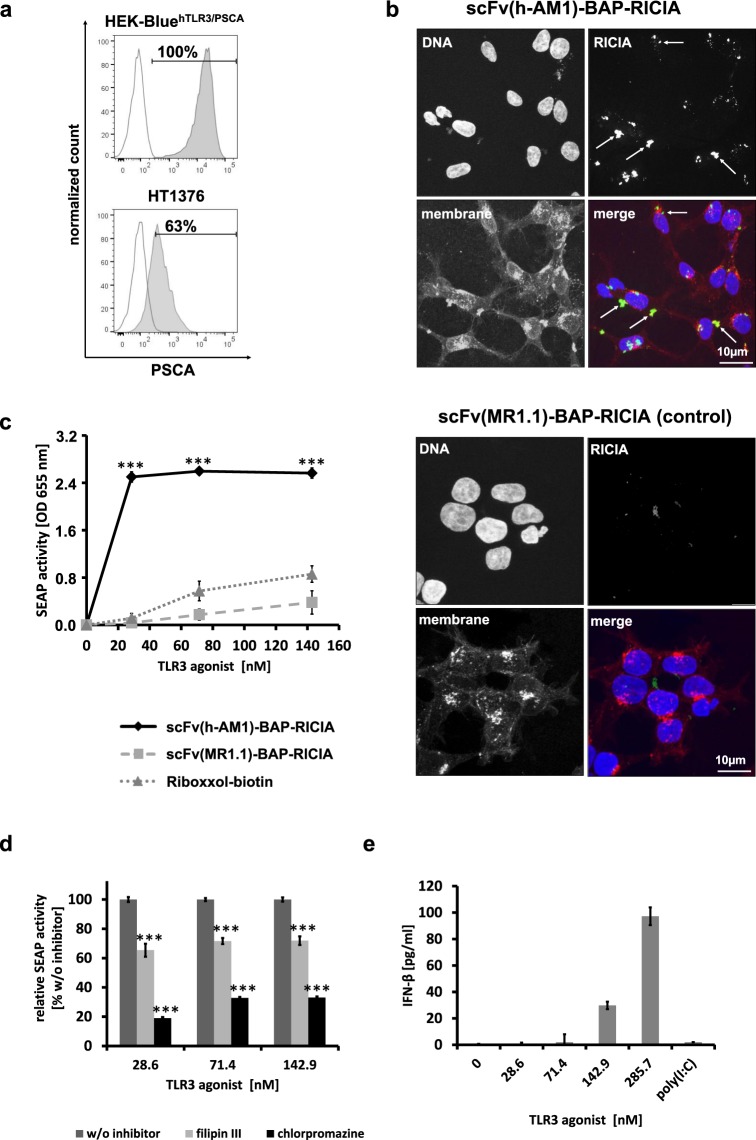
Selective delivery of scFv(h-AM1)-BAP-RICIA into PSCA-positive target cells causes NF-κB activation and subsequent release of IFN-β. (**a**) Expression levels of PSCA in HEK-Blue^hTLR3/PSCA^ reporter cell line and HT1376 cell line analyzed by flow cytometry analysis using scFv(h-AM1). (**b**) Confocal laser scanning microscopy reveals specific receptor-mediated internalization of FITC-mal19-PPI-buffered scFv(h-AM1)-BAP-RICIA into HEK-Blue^hTLR3/PSCA^ cells (see arrows) whereas no uptake is seen when using scFv(MR1.1)-BAP-conjugated control RICIA. (**c**) Treatment of HEK-Blue^hTLR3/PSCA^ with scFv(h-AM1)-BAP-RICIA resulted in the significant induction of secreted embryonic alkaline phosphatase (SEAP) activity assessed via optical density (OD) at 655 nm (mean ± SD; ***p < 0.001). (**d**) Inhibition of PSCA-mediated internalization of scFv(h-AM1)-BAP-RICIA after treatment with the inhibitors of endocytosis filipin III or chlorpromazine (mean ± SD; ***p < 0.001). (**e**) Targeted delivery of the TLR3 agonist Riboxxol formulated in scFv(h-AM1)-BAP-RICIA led to a dose-dependent release of IFN-β in HEK-Blue^hTLR3/PSCA^ (mean ± SD).

To evaluate whether the selective delivery of TLR3 agonist by RICIA induces activation of downstream signaling and type I-interferon response we generated HEK-Blue^hTLR3/PSCA^ reporter cells with ectopic expression of PSCA. Using this reporter cell line it was possible to develop immunoconjugate nanoparticles by a step-by-step assembly approach including testing of different ratios of components. Since permanent prostate cancer cell lines with endogenous PSCA expression have not been described so far, HT1376 bladder carcinoma cells, which have been identified to express robust amounts of surface PSCA, were chosen for succeeding experiments^27^. HEK-Blue^hTLR3/PSCA^ reporter cells show an improved expression of surface PSCA when compared to the HT1376 bladder cancer cells (Fig. 4a).

To assess the selective delivery of RICIA immunoconjugates we labeled the coupled dsRNA of the RICIA with FITC-mal19-PPI as described in the method section. In a previous study we have shown that the cationic glycodendrimer mal19-PPI was biocompatible, lost transfection capacity but still was competent in binding to nucleic acids^26^. Subsequent confocal laser scanning microscopy revealed that HEK-Blue^hTLR3/PSCA^ cells internalized FITC-labeled scFv(h-AM1)-BAP-containing RICIA whereas no FITC signals were detected when cells were treated with RICIA immunoconjugates coupled to scFv(MR1.1)-BAP (Fig. 4b).

Notably, 24 h treatment with scFv(h-AM1)-BAP-containing RICIA induced a dose dependent expression and secretion of the reporter SEAP by the HEK-Blue^hTLR3/PSCA^ reporter cells indicated by markedly increased OD of the cell culture medium at 655 nm indicating SEAP-mediated p-nitrophenyl phosphate substrate conversion (Fig. 4c). For the anti-PSCA-RICIA an half maximal inhibitory concentration (IC50) of 12.5 nM was calculated based on the SEAP reporter gene assay. Strikingly, treatment with scFv(MR1.1)-BAP-containing control RICIA as well as treatment with Riboxxol-biotin alone barely induced SEAP reporter gene expression. This even more indicates, the need for a cognate ligand for targeted delivery of RICIA immunoconjugates, while Riboxxol-biotin cannot easily pass cell membranes due to its net charge.

In order to address the route of the anti-PSCA-RICIA internalization in more detail, the same experiments were performed in presence of inhibitors of endocytosis. As shown in Fig. 4d the incubation of cells with chlorpromazine, which blocks clathrin-dependent endocytosis^22^, markedly inhibited SEAP activity in RICIA-treated cells. Weaker but still significant effects were observed when cells were treated with filipin III, an inhibitor of caveolae-dependent and lipid raft-mediated endocytosis^24^. Whether the targeted delivery of TLR3 into endosomes of target cells can induce a type I-interferon response was investigated by enzyme-linked immunosorbent assay. For this, HEK-Blue^hTLR3/PSCA^ cells were treated for 36 h with increasing amounts of RICIA containing concentrations of Riboxxol-biotin ranging from 0 to 285.7 nM. Also poly(I:C), a TLR3 agonist commonly used for activation of innate immune responses, was included in the experiments. As shown in Fig. 4e, the treatment of HEK-Blue^hTLR3/PSCA^ cells with increasing doses of anti-PSCA-RICIA induced a dose-dependent release of IFN-β. Interestingly, in the chosen experimental setting treatment with 50 µg/ml poly(I:C) did not result in IFN-β release by the HEK-Blue^hTLR3/PSCA^ cells. This lack of an induction of immune response in the cells might be related to the net charge of poly(I:C) which precludes diffusion through cell membranes.

**Figure 5:**
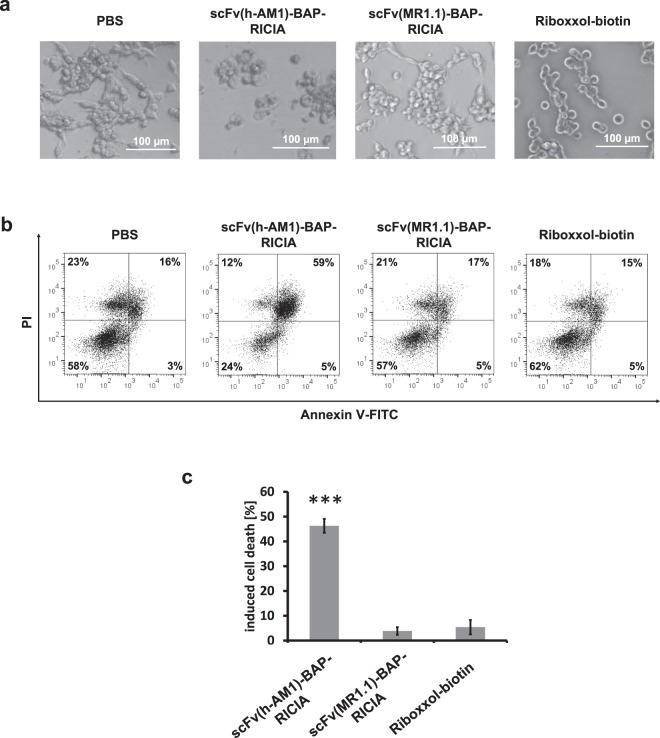
Induction of cellular apoptosis after targeted delivery of scFv(h-AM1)-BAP-RICIA in HEK-Blue^hTLR3/PSCA^ reporter cells. (**a**) Cellular morphology of RICIA- and Riboxxol-biotin-treated HEK-Blue^hTLR3/PSCA^. (**b**) scFv(h-AM1)-BAP-RICIA-induced apoptosis in HEK-Blue^hTLR3/PSCA^ cells was analyzed via Annexin V-FITC/PI staining. (**c**) Analysis of Annexin V-positive populations revealed a significant induction of cell death in scFv(h-AM1)-BAP-RICIA-treated cells (mean ± SD; ***p < 0.001).

**Figure 6:**
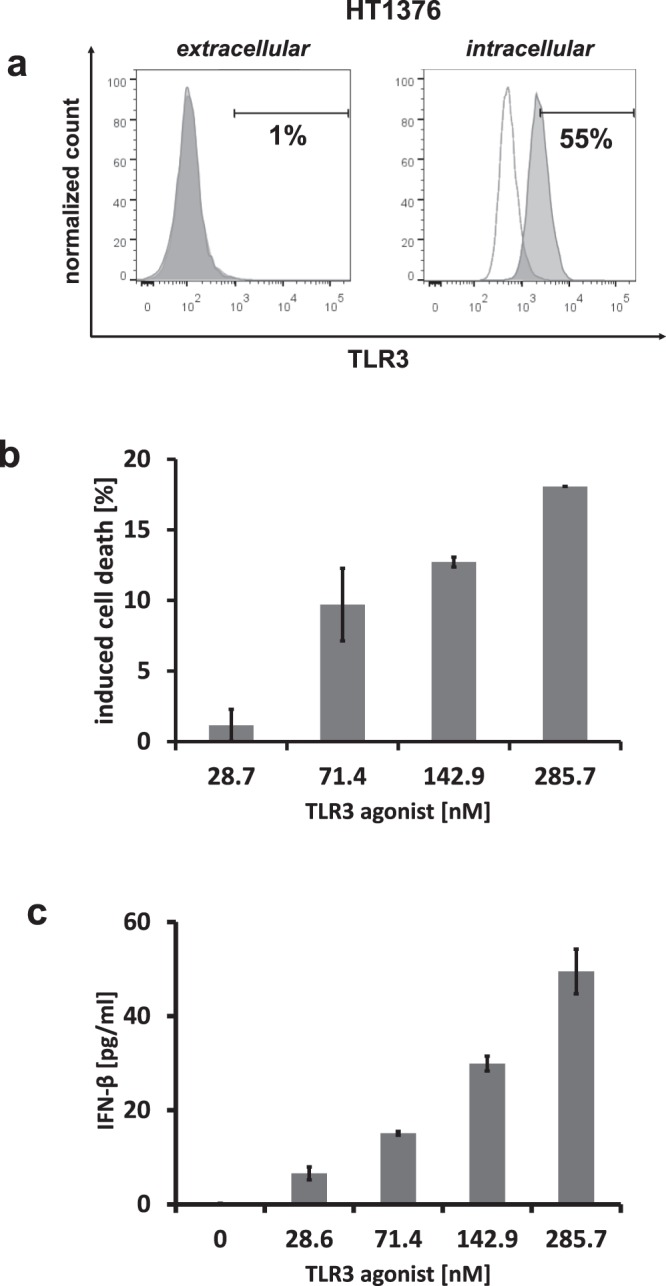
scFv(h-AM1)-BAP-RICIA treatment induces cellular apoptosis and the release of IFN-β in HT1376 cells. (**a**) Extra- and intracellular analysis of TLR3 expression of HT1376 cells. Dose-dependent induction of cellular apoptosis (**b**) and IFN-β release (**c**) after scFv(h-AM1)-BAP-RICIA treatment (mean ± SD).

Since it was of further interest whether the targeted delivery of TLR3 agonist Riboxxol affects survival of cells we performed Annexin V-FITC/PI staining of treated HEK-Blue^hTLR3/PSCA^ cells. As already seen in phase contrast microscopy depicted in Fig. 5a, cells treated with scFv(h-AM1)-BAP-containing RICIA showed dying cells and increased cellular debris which was not seen in cells treated with PBS or RICIA containing the EGFRvIII-specific scFv(MR1.1)-BAP or Riboxxol-biotin alone. When analyzing the fraction of Annexin V-positive apoptotic cells by flow cytometry it became obvious that treatment of HEK-Blue^hTLR3/PSCA^ with the scFv(h-AM1)-BAP-conjugated RICIA induced apoptosis compared to the control-RICIA and to treatment with Riboxxol-biotin alone (Fig. 5b,c). Similar results showing induction of innate immune response and apoptosis were seen when using HT1376 bladder cancer cells with endogenous expression of PSCA (see Fig. 6b,c). This cell line also endogenously expresses physiological levels of intracellular TLR3 as determined by flow cytometry analysis (Fig. 6a). HT1376 cells treated with increasing concentrations of scFv(h-AM1)-BAP-conjugated RICIA shows weaker induction of apoptosis (Fig. 6b) but an analogous robust release of IFN-β (Fig. 6c) when compared to the results obtained in HEK-Blue^hTLR3/PSCA^ reporter cells.

### **Targeted delivery of TLR3 agonist*in vivo***

**Figure 7:**
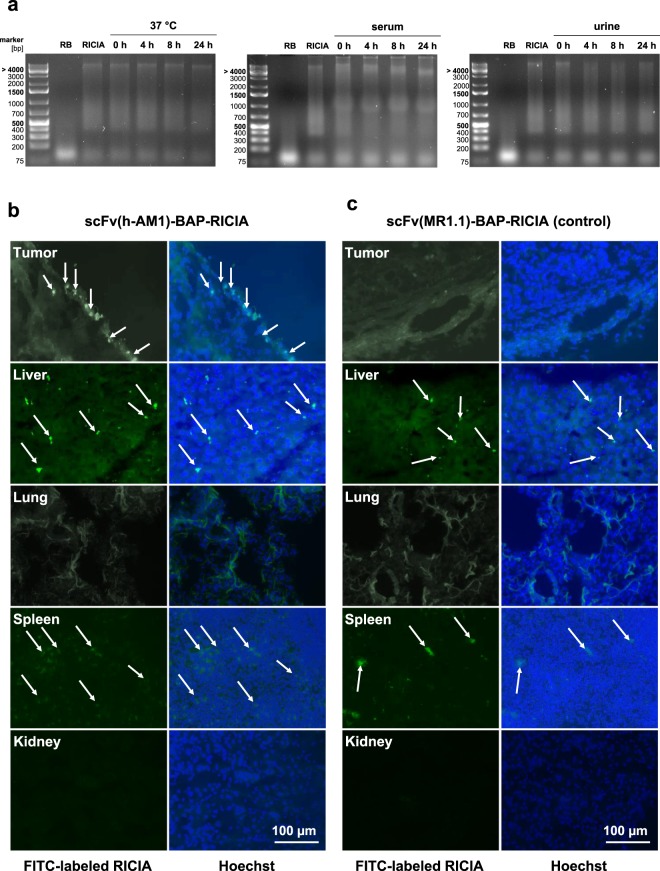
Stability of RICIA and Riboxxol and targeted delivery of scFv(h-AM1)-BAP-RICIA to PSCA-positive HT1376 bladder carcinoma xenografts. (**a**) Stability of Riboxxol-biotin and RICIA immunoconjugates at 37 °C in complexation buffer, serum and urine was analyzed in agarose gel shift assays (RB = Riboxxol-biotin). (**b**) I.p. injection of FITC-labeled scFv(h-AM1)-BAP-RICIA in NMRI^Foxnu1/Foxnu1^ mice xenotransplanted with PSCA-positive HT1376 tumors resulted in accumulation of FITC-fluorescence signals (arrows) in the tumor. (**c**) In mice injected with control scFv(MR1.1)-BAP-RICIA, no fluorescence signals were detectable in the tumor. Right panels: DNA counterstaining with Hoechst 33342 of the same cryosections. Note the appearance of a moderate and punctuated FITC uptake in liver and spleen (arrows).

In further experiments we sought to address whether the anti-PSCA-RICIA immunoconjugates can be delivered to PSCA-expressing tumors *in vivo*. First stability of Riboxxol and RICIA-bound Riboxxol-biotin in body fluids was investigated. More specifically, a mixture of Riboxxol-biotin and Riboxxol at molar ratio of 10:1 was complexed with neutravidin and scFv-BAPs and subjected to agarose gel electrophoresis. This approach allowed the simultaneous semi-quantitative assessment of Riboxxol-biotin embedded in RICIA immunoconjugates as well of non-conjugated free Riboxxol using the dsRNA-intercalating dye RedSafe. As depicted in Fig. 7a Riboxxol signals were detectable and remained unchanged after treatment with serum and urine, respectively, when compared to untreated controls. Similar results concerning stability were revealed with Riboxxol-biotin embedded in RICIAs. The RICIA immunoconjugates were not visible as distinct band but were detected as smear in the agarose gels. This result most likely reflects various nanoparticle sizes ranging from 22–55 nm and in part different net charges of immunoconjugates. Interestingly, when incubated with serum, RedSafe-stained bands >4,000 bp and between 1,000 bp and 1,500 bp were seen, which most likely indicates the development of a serum-protein corona associated with RICIA immunoconjugates. To demonstrate the capability of our scFv(h-AM1)-BAP-containing RICIA immunoconjugates to deliver Riboxxol-biotin dsRNA *in vivo*, FITC-mal19-PPI-labeled RICIA were injected intraperitoneally in NMRI^Foxn1nu/Foxn1nu^ mice xenotransplanted with HT1376 bladder cancer cells. This immune-deficient mouse strain is suitable for xenografting human tumors and was chosen for initial experiments. As depicted in Fig. 7b, the scFv(h-AM1)-BAP-conjugated RICIA were able to mark and enter tumor cells in the outer rim of the tumor specifically. In contrast, no FITC signals in tumors were observed when using EGFRvIII-specific scFv(MR1.1)-BAP-conjugated RICIA (Fig. 7c), thus indicating the specificity mediated by the targeting moiety. When using PSCA-specific RICIA as well as EGFRvIII-specific RICIA a small amount of FITC signals was detected in a punctuated pattern in liver and spleen of mice. These organs contain cells of the reticuloendothelial system such as Kupffer cells and spleen monocytes/macrophages which have been described to phagocytose several types of nanoparticles^25^. Although *in vitro* the apoptotic effect of anti-PSCA-RICIA nanoparticles on HT1376 cells was low (Fig. 6b), it was of special interest whether induction of apoptosis *in vivo* was sufficient to induce tumor growth control in HT1376 xenografted NMRI^Foxn1nu/Foxn1nu^ mice. Transplanted HT1376 tumors grew slowly and treatment of mice with three intraperitoneally injections every 48 h resulted in a diminished although statistically not significant reduction of tumor growth rates when compared to mice treated with PBS or Riboxxol-biotin alone (Supplementary Fig. 3).

## Discussion

Engagement of the innate immune system via activation of TLRs in both tumor and immune cells combined with the triggering of adaptive immune responses contains interesting prospects regarding the treatment of cancer.

In our study, we focused on TLR3 agonist Riboxxol, a 50 bp dsRNA, for immunotherapy of cancer. So far, there is accumulating evidence that activation of TLR3 in tumor cells and tumor stroma preferentially results in anti-tumor effects. For instance, treatment of human renal cancer cell lines with the synthetic TLR3 agonist poly(I:C) induced IFN-1β release and caused cell death^28^. Also treatment of human breast cancer cells with poly(I:C) has been demonstrated to induce a type I-interferon release and was accompanied by increased pro-apoptotic Bax levels and caspase-dependent apoptosis^29^. Similar results were obtained in a panel of human prostate cancer cells which after treatment with poly(I:C) succumbed to apoptosis and showed an type I-interferon response, respectively^30,31^. Interestingly, TLR3 activation in prostate cancer cell lines resulted in secretion of chemokines capable of attracting natural killer cells, granulocytes, B cells and T cells^32^. Recently, also anti-tumor effects of poly(I:C) treated *ex vivo* cultivated microglia derived from glioblastoma has been described. Notably, such (poly(I:C)) pre-activated tumor-infiltrating microglia cells were programmed into an inflammatory M1-like immune phenotype which enables them to kill glioblastoma cells and to phagocytose dying cancer cells, respectively^33^.

Yet, when considering a systemic application of natural or synthetic TLR3 agonists to treat tumor cells, the targeted delivery of TLR3 agonists appears advantageous since off-target effects are limited. Such targeted delivery of TLR3 agonists can be accomplished by implementing an antibody or specific ligand for a surface tumor antigen, which upon crosslinking is endocytosed by the tumor cell and delivers the agonist to endosomal TLR3. To reach this objective, a simple modular system for delivery of biotinylated nucleic acids, designated “Rapid Inducer of Cellular Inflammation and Apoptosis” (RICIA) was developed. For a proof-of concept we focused on the tumor-associated surface antigen PSCA and employed the novel humanized mono-biotinylated anti-PSCA scFv(h-AM1)-BAP. Noteworthy, due to RICIAs modular character the scFv-BAP antibody derivatives and nucleic acid-based TLR agonists can be easily exchanged to address tumor heterogeneity or to treat different cancers.

Notably, in our experiments we demonstrate that simultaneous binding of at least two PSCA molecules on the cells surface by a crosslinked scFv(h-AM1) antibody significantly increased endocytosis of PSCA when compared to monovalent binding of scFv(h-AM1). This also provided the basis for efficient targeted delivery by mechanisms of receptor-mediated, preferential clathrin-dependent endocytosis of scFv(h-AM1)-BAP-conjugated RICIA. The assembly of RICIA immunoconjugates was accomplished by consecutive bio-conjugation of neutravidin with mono-biotinylated scFv-BAPs and mono-biotinylated Riboxxol-biotin and resulted in nanoparticles with diameters of 22 nm to 55 nm. The unexpected formation of such nanoparticles might be due to aggregation events of the scFv-BAPs or to the formation of alternative tertiary dsRNA structures. Although not investigated so far, it appears conceivable that the nanoparticle-like format of the immunoconjugates increases avidity to PSCA-positive target cells which might further facilitate their endocytosis.

The data, obtained in the present investigation using the TLR3 agonist Riboxxol, are in line with the above mentioned promising results using poly(I:C). More specifically, anti-tumor effects including a potent induction of a type I-interferon response as well as apoptosis were observed after targeted delivery of Riboxxol to PSCA-positive tumor cells using RICIA immunoconjugates. No such effects were revealed when using RICIA conjugated to a control antibody, which demonstrates the feasibility of the targeted delivery approach. Noteworthy, treatment of HEK-Blue^hTLR3/PSCA^ reporter cells with naked poly(I:C) and Riboxxol-biotin, respectively, did not result in induction of a type I-interferon response nor in increased apoptosis (Fig. 4e and 5). Yet, in contrast to professional phagocytizing cells (i.e. macrophages, DCs), HEK-Blue^hTLR3/PSCA^ cells have only a weak phagocytic capacity which limits uptake. In addition, under physiological conditions and the chosen experimental settings poly(I:C) and Riboxxol-biotin cannot easily cross membranes due to their size and negative net charge. Therefore, previous studies utilized polycationic carrier molecules to transfect poly(I:C) into cancer cells. Such transfection of poly(I:C) and other TLR3 agonists causes disruption of endosomal membranes and result in activation of both endosomal (TLR3) and cytosolic (i.e. RIG-I, MDA5) dsRNA receptors^31^. We suggest a predominant TLR3 activation utilizing receptor-mediated endocytosis for delivery of Riboxxol. However, additional activation of cytosolic dsRNA receptors due to leakage or disruption of endosomal membranes upon RICIA-treatment cannot be fully ruled out. However, this might be advantageous since it could further improve induction of a type I-interferon response and apoptosis in targeted cancer cells.

In line with a previous report addressing the stability of a similar type of dsRNA^14^, Riboxxol, when assembled in RICIA, remains stable when treated with serum or urine. When administered intraperitoneally in nude mice, anti-PSCA-RICIA specifically accumulated in xenografted HT1376 tumors, whereas the treatment with EGFRvIII-specific RICIA, which were used as negative control, did not deliver Riboxxol to PSCA-positive tumors (Fig. 7b,c). Noteworthy, the *in vivo* experiments showed no anti-PSCA- as well as anti-EGFRvIII-RICIA signals in lung and kidneys and only some weak punctuated signals in liver and spleen. In the latter organs this likely is due to phagocytizing cells of the reticuloendothelial system (i.e. spleen monocytes/macrophages, Kupffer cells of the liver). Collectively, the data show that TLR3 agonist Riboxxol can be selectively delivered to xenografted tumor cells expressing the cognate surface receptor *in vivo* by assembled RICIA immunoconjugates. Of note, there is only 60% homology of human and mouse PSCA protein and the humanized anti-PSCA scFv(h-AM1) does not bind to murine PSCA (data not shown). When considering treatment of patients suffering from PSCA-positive cancers with anti-PSCA-RICIA, it can be predicted that tumorigenic as well as normal cells expressing PSCA at sufficient surface levels and containing TLR3 are affected by anti-PSCA-RICIA. It remains to be investigated whether on-target/off-tissue effects are limited in normal epithelial cells having predominant PSCA expression at the apical cell membrane. Otherwise, in de-differentiated epithelial cancer cells displaying a more de-polarized cell type, PSCA might be more accessible for anti-PSCA-RICIA. However, from a general point of view on-target/off-tissue effects affecting the prostate or other PSCA-positive normal tissues or cell types cannot be excluded and must be addressed in proper risk assessment. In our study, the observed anti-tumoral effects, in particular the induction of a type I-interferon response, after targeted delivery of Riboxxol substantiates TLR3 agonists as promising candidates for cancer immunotherapy. Yet, efficacy of anti-PSCA-RICIA in immune-deficient mice bearing PSCA-positive human xenografts was limited (Supplementary Fig. 3). Therefore, future experiments using syngeneic mouse tumor models are needed to address a possible induction of adaptive immune responses and to assess on-target/off-tissue effects when applying anti-PSCA-RICIA.

## Methods

### Cell lines

The human embryonic kidney cell lines HEK293T, HEK293T^PSCA^ and HEK293T^EGFRvIII^ have been described previously^26^ and were cultured in DMEM complete with 4.5 g/l glucose, 10% v/v heat-inactivated FCS, 100 U/ml penicillin, 100 µg/ml streptomycin and 10 mM HEPES (all from Life Technologies). HEK293T^huBirA^ cells stable expressing codon-optimized biotin ligase huBirA^26^ were maintained in DMEM complete supplemented with 50 µM Biotin-C6 (Sigma-Aldrich). Human FreeStyle™ 293-F cell line (Invitrogen) was maintained in special FreeStyle™ 293 Expression Medium (Life Technologies). Human HEK-Blue^hTLR3^ (Invivogen) and HEK-Blue^hTLR3/PSCA^ SEAP reporter cell lines expressing the human TLR3 gene were cultured in DMEM with 4.5 g/l glucose, 10% v/v heat-inactivated FCS, 100 µg/ml normocin, 30 µg/ml blasticidin and 100 µg/ml zeocin (all antibiotics from Invivogen). The bladder cancer cell line HT1376 with endogenous expression of PSCA was maintained in DMEM complete supplemented with 1X non-essential amino acids (Life Technologies). FreeStyle™ 293-F cells were incubated at 37 °C and 8% CO_2_ and all other cell lines with 5% CO_2_ in a humidified incubator_._

### Development of humanized scFv(h-AM1)

The humanized scFv(h-AM1) was designed *in silicio* by engrafting the CDRs of the murine scFv(AM1) into framework regions of human Ig germline genes. The CDRs of the murine scFv(AM1) were identified using an algorithm described by North *et al*.^34^ and were used to identify suitable human Ig germline genes by alignment with the software “IgBLAST” (NIH)^35,36^. CDRs of scFv(AM1)-V_L_ were engrafted into the IGKV1-39*01 germline gene. Since no suitable framework region was identified for the C-terminus of scFv(AM1)-V_H_, the V_H_ was only partially humanized by engrafting CDR1 and CDR2 into the IGHV3-23*03 germline gene. In an additional step the partially humanized scFv(AM1)-V_H_ was engrafted into the IGHV1-NL1*01 germline gene resulting in a fully humanized scFv(AM1)-V_H_ containing framework regions from IGHV3-23*03 and IGHV3-23*03.

### Vector constructs

DNA encoding anti-PSCA scFv(h-AM1) fused to a N-terminal Igκ leader sequence, to C-terminal c-myc epitope and 6xHis tag^37^ and with or without short 22 amino acid biotin acceptor peptide (BAP), were synthesized (Eurofins MWG Operon) and ligated into the lentiviral pHATtrick-PuroR^27^ vector resulting in pHATtrick-scFv(h-AM1)-PuroR and pHATtrick-scFv(h-AM1)-BAP-PuroR vectors. EGFRvIII-specific control antibody derivatives scFv(MR1.1) and scFv(MR1.1)-BAP were produced accordingly, resulting in pHATtrick-scFv(MR1.1)-PuroR and pHATtrick-scFv(MR1.1)-BAP-PuroR vectors. All vectors were verified by DNA sequencing.

### Lentiviral transduction

Transduction of scFv-encoding vectors was accomplished using a transient lentiviral packaging system^38^. Briefly, HEK293T cells were transiently transfected with the packaging vectors pCD/NL-BH^39^ and pcz-VSV-G^40^ as well as the scFv-encoding lentiviral vector using polyethyleneimine (PEI). Production of viral particles was enhanced with 10 µM sodium butyrate 12 h after transfection. Medium was changed after 8 h and lentiviral supernatant was harvested 12 h later, filtered with a 0.45 µm pore size filter and supplemented with 8 µg/ml polybrene (Sigma-Aldrich). Transduction of 1.5 × 10^5^ HEK293T^huBirA^ or FreeStyle™ 293-F cells was performed in a 6-well with 2 ml of lentiviral supernatant and repeated after 24 h. Transduced cells were selected with 15 µg/ml puromycin for 24 h. HEK-Blue^hTLR3^ reporter cell line was engineered to express PSCA using the vector p6NST53-PSCA as described previously^27^. Transduced cells were designated HEK-Blue^hTLR3/PSCA^ and selected with 100 µg/ml zeocin for one week.

### Production and purification of scFvs

Biotinylated scFv-BAPs were produced by the generated HEK293T^huBirA/scFv(h-AM1)-BAP^ and HEK293T^huBirA/scFv(MR1.1)-BAP^ cell lines whereas parental scFvs were expressed by the generated FreeStyle™ 293-F^scFv(h-AM1)^ and 293-F^scFv(MR1.1)^ cells. All scFvs were directly purified from supernatants using a Ni^2+^-NTA affinity chromatography. Briefly, 25 ml of clarified supernatant was passed through Ni^2+^-NTA spin column (Qiagen) and washed with 1X PBS containing 150 mM NaCl and 10 mM imidazole and afterwards 20 mM imidazole. Column bound scFvs were eluted in 500 µl fractions using 1X PBS containing 150 mM NaCl and 350 mM imidazole. Eluted scFvs were dialyzed in 1X PBS twice for 2 h and additionally for 12 h at 4 °C. Biotinylated scFv-BAPs were further purified using a monomeric avidin kit (Pierce). Eluates were dialyzed with 1X PBS as described previously. Biotinylated scFv-BAPs were subsequently concentrated using Amicon tubes Ultra-15 (10 kDa exclusion size; Merck Millipore).

### Flow cytometry

Specific binding of scFvs and scFv-BAPs to appropriate antigen or investigation of PSCA expression on target cells was assessed staining 2 × 10^5^ cells with 5 µg scFv or scFv-BAP for 1 h at 4 °C and subsequent with secondary anti-c-myc-FITC antibody (Miltenyi) or for investigation of successful biotinylation of scFv-BAPs with secondary anti-Biotin-PE antibody (Miltenyi). For internalization studies scFv(h-AM1)-stained HEK293T^PSCA^ cells were incubated for 2, 4, 6, 8, 24, and 48 h with anti-c-myc-Biotin (Miltenyi) resulting in crosslinked PSCA. For monovalent binding scFv(h-AM1)-stained HEK293T^PSCA^ were incubated after above-named incubation times with anti-c-myc-Biotin. PSCA expression was subsequently detected with anti-Biotin-PE. Staining with secondary antibody only was included in all experiments as a control. TLR3 expression in HT1376 cells was confirmed using a goat polyclonal TLR3 antibody (N-14, Santa Cruz Biotechnology) and a secondary donkey anti-goat IgG-FITC antibody (Jackson ImmunoResearch). Appropriate IgG isotype was used as control. Apoptosis assay were performed by Annexin V-FITC/PI kit (Miltenyi) after treatment of either 5 × 10^4^ HEK-Blue^hTLR3/PSCA^ cells with RICIAs based on 1 µg/ml (28.57 nM) Riboxxol-biotin, Riboxxol-biotin only or PBS or of HT1376 cells with RICIA based on 1 µg/ml (28.6 nM), 2 µg/ml (71.4 nM), 5 µg/ml (142.9 nM), 10 µg/ml (285.7 nM) Riboxxol-biotin or PBS for 24 h. For calculation of induced cell death percentage of Annexin V-positive PBS-treated cell fraction was subtracted from percentage of RICIA- or Riboxxol-biotin-treated cell fractions. Fluorescence signals were measured using a MACSQuant flow cytometer (Miltenyi) and analyzed with FlowJo software version 7.6.5. (Tree Star).

### Western blot analysis

Purity of scFvs was determined by SDS-PAGE with 2.5–5 µg protein and subsequent staining of proteins with SimplyBlue^TM^ SafeStain (Invitrogen). For immunoblot analysis 1.5 µg of scFv were separated by SDS-PAGE electrophoresis under reducing conditions. After protein transfer to a PVDF membrane (Whatman), scFvs were detected using a primary mouse anti-c-myc antibody (Invitrogen) and a secondary polyclonal rabbit anti-mouse IgG HRP conjugate (Dako). Biotinylated scFv-BAPs were detected with a HRP-conjugated anti-biotin antibody (Sigma-Aldrich). Visualization of scFvs was performed by Luminata Forte Western HRP substrate (Merck Millipore) and LAS 3000 chemoluminescence imager (FujiFilm). For analysis of scFv-BAP complexation with avidin, the two components were mixed at various molar ratios ranging from 1:2 to 8:1 in PBS. After 30 min incubation at room temperature scFv-BAP-avidin conjugates as well as non-conjugated biotinylated scFv-BAPs were analyzed by non-reducing SDS-PAGE and detected by Western blot analysis as described above.

### Assembly and functional analysis of RICIA

RICIA were generated in the first step by conjugation of biotinylated scFv-BAPs with neutravidin (Pierce) for 30 min in PBS and in a second step with TLR3 agonist Riboxxol-biotin (riboxx life science) for 30 min in complexation buffer (10 mM HEPES, 150 mM NaCl, pH 7.4, Fig. 3b) in a final molar ratio of 2:1:2. For functional analysis of RICIA HEK-Blue detection system according to the manufacturer’s instructions (Invivogen) was used. Briefly, RICIA based on 1 µg/ml (28.6 nM), 2.5 µg/ml (71.4 nM) and 5 µg/ml (142.9 nM) Riboxxol-biotin or Riboxxol-biotin only were incubated with 5 × 10^4^ HEK-Blue^hTLR3/PSCA^ cells for 24 h. In another experiment 0.3 µg/ml filipin III or 3 µg/ml chlorpromazine (both Sigma-Aldrich) was added to block caveolae- and chlatrin-mediated endocytotic pathways, as described previously^26^. Hydrolysis of substrate by SEAP was quantified at 655 nm by Synergy 2 Multi-Mode Microplate Reader (BioTek Instruments). Values of endocytotic pathway analysis were normalized to untreated cells. To investigate the stability of RICIA immunoconjugates as well as of Riboxxol itself in body fluids, RICIA were generated based on 2 µg Riboxxol-biotin/Riboxxol (10:1) and incubated with either 50% serum or urine or in complexation buffer at 37 °C. RICIA and Riboxxol-biotin controls were stored at 4 °C. The stability after 0 h, 4 h, 8 h, and 24 h was assessed via agarose gel shift assays. After adding 1X DNA gel loading dye (Thermo Fisher Scientific) and subsequent electrophoresis, fluorescence of RedSafe (iNtRON Biotechnology)-stained RNA was detected using the UV transilluminator AlphaImager (AlphaInnotech).

### Atomic force microscopy (AFM)

For *in-situ* AFM, silicon wafers were treated with O_2_-plasma to obtain a hydrophilic surface for the adsorption of RICIA based on 1 µg/ml Riboxxol-biotin. The AFM measurements in fluids were done in the peak force tapping mode by a Dimension ICON (Bruker-Nano). Silicon nitride sensors SCANASYST-FLUID+ (Bruker-Nano) with a nominal spring constant of 0.7 N/m^−1^ and a nominal tip radius of 2 nm were used. The particle size distribution was calculated by the software NanoScope Analysis (Bruker-Nano).

### IFN-β ELISA

To investigate TLR3-induced type I-interferon response, 5 × 10^4^ HEK-Blue^hTLR3/PSCA^ or HT1376 cells were stimulated with scFv(h-AM1)-BAP-RICIA based on 1 µg/ml (28.6 nM), 2 µg/ml (71.4 nM), 5 µg/ml (142.9 nM), 10 µg/ml (285.7 nM) Riboxxol-biotin or with 50 µg/ml poly(I:C) for 36 h. Collected supernatants were analyzed using VeriKine Human IFN Beta ELISA Kit (pbl Assay Science). Quantification of IFN-β concentration was performed in triplicates at 450 nm by Synergy 2 Multi-Mode Microplate Reader (BioTek Instruments) and calculated using an IFN-β standard curve.

### Confocal laser scanning microscopy

Cellular uptake of RICIA was analyzed using 2.5 × 10^5^ HEK-Blue^hTLR3/PSCA^ cells and FITC-labeled RICIA. FITC-labelling of RICIA was accomplished by complexing with maltose-modified 4^th^ generation FITC-poly(propyleneimine)-dendrimer (FITC-mal19-PPI) at a molar ratio of 1:2.5. Cells were treated with FITC-stained-RICIA for 24 h in DMEM complete medium containing FCS. Afterwards cells were fixed, stained (cell membrane with Texas Red-X conjugate of WGA and DNA with Hoechst 33342) and analyzed by confocal laser scanning microscope Leica SP5.

### Delivery of RICIA to bladder cancer xenografts

The animal experiments were approved by the Landesdirektion Sachsen (animal experiment permission number: DD24-5131/354/14) and the methods were carried out in accordance with the approved guidelines and regulations. 2 × 10^6^ HT1376 cells were subcutaneously implanted in the right flank of athymic female nude mice (NMRI^Foxn1nu/Foxn1nu^). After reaching an average tumor diameter of 5–8 mm, mice were injected intraperitoneally with FITC-labeled mal19-PPI-RICIA, described above, containing 5 µg (142.9 nM) Riboxxol-biotin. Tumor, liver, lung, spleen, and kidney were resected 18 h after injection and 10 µm thick cryosections were prepared. After staining of DNA with Hoechst 33342, the slides were analyzed by Axio Examiner Z1 fluorescence microscope (Zeiss). For treatment with anti-PSCA-RICIA, HT1376 xenografts were established as described above and groups of animals with similar mean tumor volumes were treated with three intraperitoneal injections of PBS, 142.9 nM Riboxxol-biotin in PBS or anti-PSCA-RICIA containing 142.9 nM Riboxxol in total volumes of 200 µl at days 1, 3 and 5. Tumor sizes were measured in both directions by a digital caliper every two days. After calculating the mean of the tumor diameter (d), the volume of each tumor was calculated by the formula V_Tumor_ = 1/6 × Π × d^3^.

### Statistical analysis

All experiments were performed at least three times. Differences between groups were examined for statistical significance using Student’s *t*-test. Values of p < 0.05 were considered as statistically significant: *p < 0.05, **p < 0.01, ***p < 0.001.

## Supplementary information

Datasets 1-4

## Data Availability

Data will be provided upon reasonable request.
