# Supplementary material for: Targeted delivery of TLR3 agonist to tumor cells with single chain antibody fragment-conjugated nanoparticles induces type I-interferon response and apoptosis
Source: Sci Rep. 2019 Mar 1;9:3299. doi: 10.1038/s41598-019-40032-8 (PMC6397204; doi:10.1038/s41598-019-40032-8)

Supplementary material for the manuscript:

Targeted delivery of TLR3 agonist to tumor cells with single chain antibody fragment-conjugated nanoparticles induces type I-interferon response and apoptosis

Isabell Schau[1], Susanne Michen[1*], Alexander Hagstotz[1], Andreas Janke[2], Gabriele Schackert[1,3], Dietmar Appelhans[2], and Achim Temme[1,3*]

[1]Department of Neurosurgery, Section Experimental Neurosurgery and Tumor Immunology, University Hospital Carl Gustav Carus, TU Dresden, Fetscherstraße 74, 01307 Dresden, Germany

[2]Leibniz Institute of Polymer Research Dresden e.V., Mailbox 120411, 01069 Dresden, Germany

[3]German Cancer Consortium (DKTK), Dresden, Germany; German Cancer Research Center (DKFZ), Heidelberg, Germany

*Corresponding authors

Emails: achim.temme@uniklinikum-dresden.de, susanne.michen@uniklinikum-dresden.de

Figure and Table Legends

Supplementary Figure 1: Accompanying full-length Coomassie-stained polyacrylamide gels and immunoblots for Figure 1b (a) and 1c (b).

Cropping of images is indicated with dotted lines. scFv(h-AM1): open arrowhead, scFv(h-AM1)-BAP: black arrowhead, M: marker.

Supplementary Table 1: Humanization of scFv(AM1) increases binding affinity to PSCA.

Supplementary Figure 2: Amino acid sequence of murine scFv(AM1) and humanized scFv(h-AM1).

V_H- and V_L-sequences are written in green or purple. CDRs are indicated in grey and amino acid substitutions due to humanization in red. Potential O-glycosylation sites are marked by asterisks and were calculated by NetOGlyc 4.0 Server (Steentoft, C *et al.* Precision mapping of the human O-GalNAc glycoproteome through SimpleCell technology. *EMBO J* **32(10)**,1478-88, (2013)).

Supplementary Figure 3: Effects of anti-PSCA-RICIA treatment on growth of PSCA-positive HT1376 tumors xenotransplanted in NMRI$^{Foxnu1/Foxnu1}$ mice.

Tumor growth over 10 days of repetitively (day 1, 3 and 5, black arrows) anti-PSCA-RICIA treated mice in comparison to control mice injected with PBS (a) or Riboxxol-biotin (b) alone.

Supplementary Figure 1

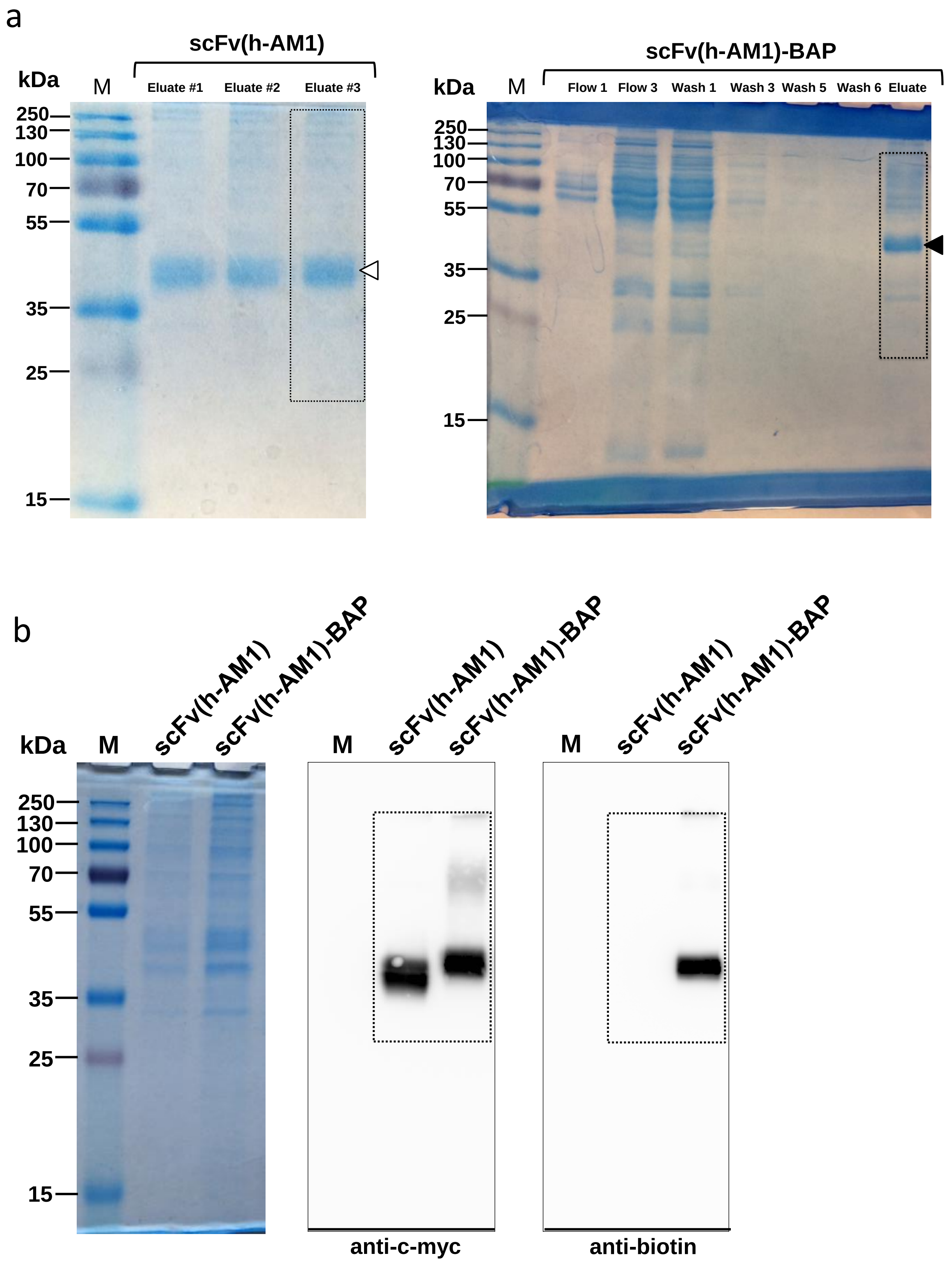

Supplementary Table 1

scFv	dissociation constant K_D [M] ± SE
scFv(h-AM1)-BAP	$8.3 \times 10^{-7} \pm 1.6 \times 10^{-7}$
scFv(AM1)-BAP	$1.7 \times 10^{-5} \pm 1.6 \times 10^{-5}$

Supplementary Figure 2

Murine scFv(AM1)

NH_2 - Igκ signal peptide – GS**QVKLQESGGGLVQPGGSLKLSCVASGFTFSSYTMSWVRRTPEKRL**

EWVAYIHNGGGHTYYPDTIKGRFTISRDNAKNTLFLEMSSLKSEDTAMYYCTRRMYYGNSHWYF

* ** ** * *

DVWGAGTSVTVSSAKTTPPSVYGGGGSGGGGSGGGGSTNS**DIVMTQSPSSLSASLGDRVTINCR**

TSQDISNYLNWYQLTPDGTVKLLIYYTLKLNSGVPSRFSGSGSGTDYSLTINNLEKEDFATYFC

QQSKTLPWTFGGGTKLEIKRA - c-myc epitope - 6xHis tag - COOH

Humanized scFv(h-AM1)

NH_2 - Igκ signal peptide – GS**EVQLLESGGGLVQPGGSLRLSCVASGFTFSSYTMSWVRQAPGKGL**

EWVSYIHNGGGHTYYADSVKGRFTISRDNSKNTLYLQMNSLRAEDTAVYYCTRRMYYGNSHWYF

* ** ** *

DVWGAGTRVTITSAKTTPPSVYGGGGSGGGGSGGGGSTNS**DIQMTQSPSSLSASVGDRVTITCR**

TSQSISNYLNWYQQKPGKAPKLLIYYTLKLNSGVPSRFSGSGSGTDFTLTISSLQPEDFATYYC

QQSKTLPWTFGGGTKLEIKRA - c-myc epitope - 6xHis tag - COOH

Supplementary Figure 3

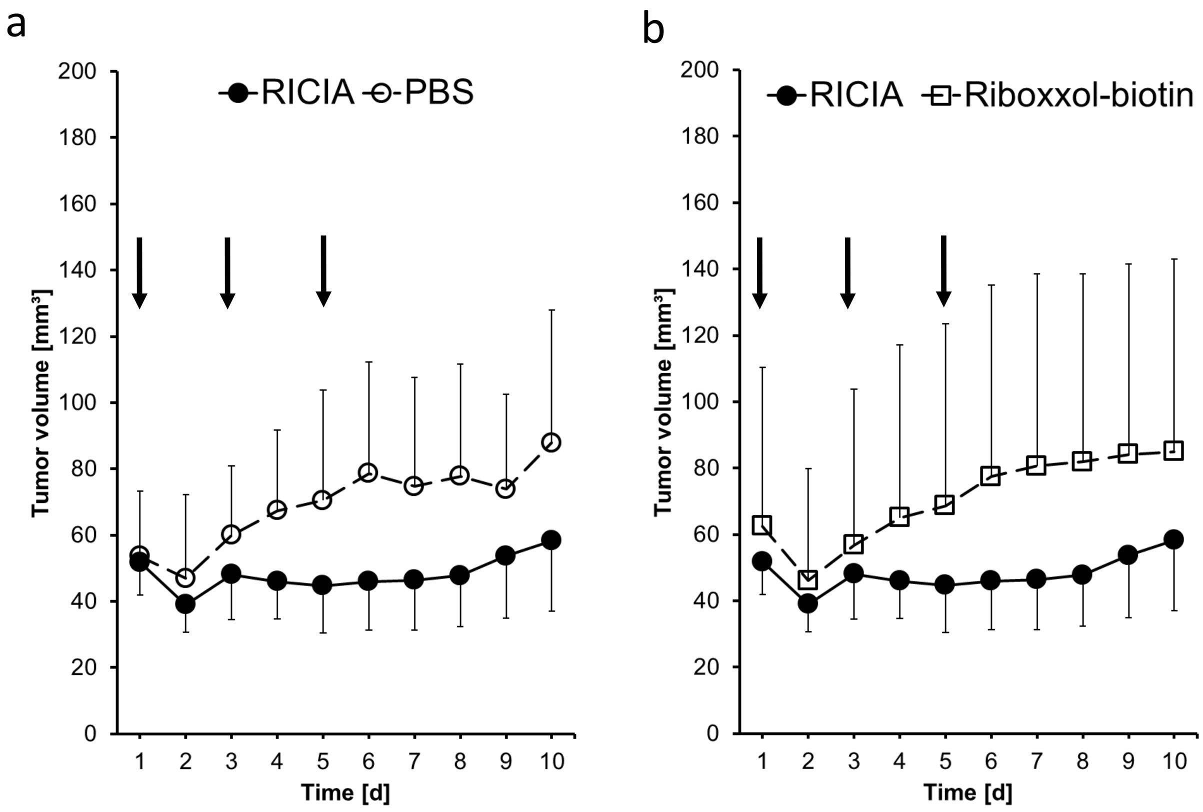

Supplement: Supplementary file 1 — Datasets 1-4 [file 41598_2019_40032_MOESM1_ESM.pdf]
